# Serum IL‐36 cytokines levels in type 2 diabetes mellitus patients and their association with obesity, insulin resistance, and inflammation

**DOI:** 10.1002/jcla.23611

**Published:** 2020-10-09

**Authors:** Yan Li, Sisi Chen, Tingqi Zhao, Mingcai Li

**Affiliations:** ^1^ Department of Immunology Zhejiang Key Laboratory of Pathophysiology Ningbo University School of Medicine Ningbo China; ^2^ Department of Endocrine The Affiliated Hospital of Ningbo University School of Medicine Ningbo China

**Keywords:** C‐reactive protein, interleukin‐17, interleukin‐36, low‐density lipoprotein, pro‐inflammatory cytokines, type 2 diabetes mellitus

## Abstract

**Background:**

The interleukin (IL)‐36 cytokines include IL‐36α, IL‐36β, IL‐36γ, and IL‐36Ra. Little was known about their roles in type 2 diabetes mellitus (T2DM).

**Methods:**

The study included 40 T2DM patients and 42 healthy control subjects. The anthropometric and biochemical measurements were performed using automatic biochemical analyzer, high‐performance liquid chromatography, and electrochemiluminescence immunoassay. Circulating IL‐36α, IL‐36γ, IL‐36Ra, and IL‐17 levels were determined by enzyme‐linked immunosorbent assay.

**Results:**

Serum IL‐36α, IL‐36γ, and IL‐17 levels in T2DM patients were significantly higher than those in controls, whereas serum IL‐36Ra levels in T2DM patients were lower. Correlation analysis showed that serum IL‐36α was positively correlated with high sensitivity C‐reactive protein. Serum IL‐36α was negatively correlated with IL‐36Ra. Serum IL‐17 was negatively correlated with low‐density lipoprotein cholesterol.

**Conclusions:**

This study demonstrated that T2DM patients displayed increased IL‐36α and IL‐36γ expression and decreased IL‐36Ra expression. Moreover, the inflammatory cytokine levels were directly proportional to the inflammation and blood lipid levels. Our results suggest that IL‐36 cytokines may be a new target for the diagnosis or treatment of T2DM.

## Graphical abstract

The IL‐36α, IL‐36γ, and IL‐17 levels in the T2DM patients were significantly higher than those in the healthy individuals. Conversely, the serum IL‐36Ra level of T2DM patients was significantly lower than of the control subjects. Pearson correlation analysis of serum IL‐36 cytokines levels and clinical biochemical parameters in T2DM patients showed that IL‐36α was positively correlated with hsCRP. IL‐17 was negatively correlated with LDL‐C.

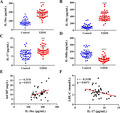

## 1 INTRODUCTION

Type 2 diabetes mellitus (T2DM) accounts for 90% of global diabetes and has a high mortality. T2DM is related to insulin resistance (IR) and chronic inflammation in white adipose tissue. Adipose tissue can release a variety of pro‐inflammatory cytokines. Many pro‐inflammatory cytokines and chemokines have been identified to play an important role in chronic inflammation and IR caused by obesity, including interleukin (IL)‐1β, IL‐6, tumor necrosis factor (TNF)‐α, and monocyte chemoattractant protein (MCP)‐1.^1^

IL‐36 cytokines belong to the IL‐1 family (IL‐1F), including three agonists (IL‐36α, IL‐36β, IL‐36γ) and a natural IL‐36 receptor antagonist (IL‐36Ra). Because the IL‐36 cytokines have a high homology with IL‐1β and IL‐1Ra, respectively, of the IL‐1F, they are classified into the IL‐1F and named IL‐1F6, IL‐1F8, IL‐1F9, and IL‐1F5. With the study of the function of these newly discovered IL‐1F members and the identification of their homologous IL‐1 receptor‐related protein 2 (IL‐1Rrp2), IL‐1F6, IL‐1F8, IL‐1F9, and IL‐1F5 were renamed and classified as the IL‐36 family, namely, IL‐36α (IL‐1F6), IL‐36β (IL‐1F8), IL‐36γ (IL‐1F9), and IL‐36Ra (IL‐1F5) in 2010.^2^ Additionally, IL‐1Rrp2 was renamed IL‐36 receptor (IL‐36R). Similar to IL‐1α and IL‐1β, IL‐36α, IL‐36β, and IL‐36γ bind to IL‐36R and co‐receptor IL‐1R accessory protein (IL‐1RAcP) to activate the nuclear transcription factor kappa B (NF‐κB) and mitogen‐activated protein kinase signaling pathways. IL‐36Ra has a similar effect to IL‐1Ra and inhibits the activation of these pathways by binding to IL‐36R.^3^

Recent studies found that IL‐36 cytokines play a key role in obesity‐related metabolic disorders, low‐grade inflammation, and IR. Several studies showed that IL‐36 cytokines have effects on psoriasis, arthritis, and systemic lupus erythematosus, but there are few reports on the IL‐36 cytokines in T2DM.^4^, ^5^ To explore the clinical relevance of IL‐36 cytokines in T2DM, we determined the serum IL‐36 cytokines concentrations of normal subjects and T2DM patients, and analyzed their relationships with IR, anthropometry, and metabolic parameters.

## 2 MATERIALS AND METHODS

### 2.1 Study design and participants

Forty patients diagnosed with T2DM were recruited from the Affiliated Hospital of Ningbo University School of Medicine (Ningbo, China). T2DM diagnosis was based on the 75 g oral glucose tolerance test and met the diagnostic criteria in the Guidelines for the Prevention and Treatment of T2DM in China (2017 Edition). Additionally, 42 healthy individuals with age and gender matching undergoing regular physical examination were recruited as a control group. Exclusion criteria included T1DM, acute or chronic complications, hypertension, or organ failure. The study design was approved by the Ethics Committee of Ningbo University School of Medicine. All subjects gave their informed consent.

### 2.2 Anthropometric and biochemical measurements

Standard methods for anthropometric measurements, including weight, height, and calculated body mass index (BMI), were applied to all subjects. Appointed nurses used a mercury sphygmomanometer to determine systolic blood pressure (SBP) and diastolic blood pressure (DBP). The subjects were fasted overnight and blood samples collected from 8 to 9 am the next day. The blood samples were kept at room temperature for 2 hours, centrifuged at 1000 *g* for 20 minutes, and the supernatant was taken aseptically and stored at −80°C.

An automatic biochemical analyzer (AU5800; Beckman) was used to determine the triglyceride (TG), total cholesterol (TC), high‐density lipoprotein cholesterol (HDL‐C), low‐density lipoprotein cholesterol (LDL‐C), high sensitivity C‐reactive protein (hsCRP), and fasting blood glucose (FBG) levels. Glycated hemoglobin (GHb) was measured by high‐performance liquid chromatography. The fasting insulin (FINS) concentration was determined using an electrochemiluminescence immunoassay. The IR was estimated using homeostasis model assessment of insulin resistance (HOMA‐IR):

HOMA‐IR = FINS (μU/mL) × FBG (mmol/L)/22.5.

### 2.3 Measurements of IL‐36 and related cytokines

Enzyme‐linked immunosorbent assay was used to determine the serum IL‐36α, IL‐36γ, IL‐36Ra, and IL‐17 levels. The kits were purchased from Cusabio Biotech Co., Ltd. The experimental procedures followed the manufacturer's instructions. All samples were assayed in duplicate and random order.

### 2.4 Statistical analysis

Data were expressed as mean ± SD. The two groups were compared by two‐tailed unpaired Student's *t* test. Pearson correlation analysis was performed to assess the correlation between IL‐36 cytokines and variables. Statistical analyses were performed using GraphPad Prism 7 (GraphPad Software Inc). Values of *P* < .05 were considered statistically significant. *P < .05, **P < .01, ***P < .001, ****P < .0001.

## 3 RESULTS

### 3.1 Characteristics of study participants

Table 1 summarizes the anthropometric indexes of the research subjects. There were no statistically differences in age, height, weight, DBP, or gender distribution between the T2DM patients and the healthy controls (*P* > .05). Conversely, the BMI and SBP of the T2DM patients were statistically significantly higher than those of the control subjects (*P* < .05 and *P* < .0001, respectively).

**Table 1:** Comparison of general information of the research subjects

Parameters	Healthy control subjects (42)	T2DM patients (40)	*P*
Gender			
Male	25	24	
Female	17	16	.5110
Age (year)	55.4 ± 8.80	57.1 ± 9.40	.4012
Height (m)	1.68 ± 1.21	1.65 ± 2.32	.0828
Weight (kg)	63.36 ± 2.13	65.37 ± 2.62	.3410
BMI (kg/m^2^)	22.28 ± 0.32	23.85 ± 0.52	.0114*
SBP (mm Hg)	119.1 ± 1.67	134.4 ± 3.26	<.0001****
DBP (mm Hg)	76.83 ± 1.25	76.58 ± 1.67	.9013

Abbreviations: BMI, body mass index; DBP, diastolic blood pressure; SBP, systolic blood pressure; T2DM, type 2 diabetes mellitus.

*, **** Statistically significant between the T2DM and control subjects.

### 3.2 The clinical biochemical parameters of the subjects

The GHb, CRP, LDL‐C, FBG, FINS, and HOMA‐IR levels of patients in the T2DM patients were statistically significantly higher than those of the control subjects (Table 2). Conversely, the HDL‐C, TC, and TG contents were not statistically different between the two groups (*P* > .05).

**Table 2:** Clinical biochemical parameters

Variables	Controls (42)	T2DMs (40)	*P*
TC (mmol/L)	4.659 ± 0.12	4.808 ± 0.22	.5506
TG (mmol/L)	1.547 ± 0.13	1.754 ± 0.21	.4036
LDL‐C (mmol/L)	2.122 ± 0.09	2.551 ± 0.15	.0142*
HDL‐C (mmol/L)	1.274 ± 0.04	1.218 ± 0.06	.4506
FBG (mmol/L)	4.703 ± 0.06	10.10 ± 1.33	<.0001****
FINS (μU/mL)	8.573 ± 0.67	16.65 ± 2.09	.0003***
hsCRP (mg/L)	3.292 ± 0.47	11.68 ± 0.81	<.0001****
GHb (%)	7.277 ± 0.14	10.51 ± 0.43	<.0001****
HOMA‐IR	1.799 ± 0.14	7.888 ± 1.74	.0006***

Abbreviations: FBG, fasting blood glucose; FINS, fasting insulin; GHb, glycated hemoglobin; HDL‐C, high‐density lipoprotein cholesterol; HOMA‐IR, homeostasis model assessment of insulin resistance; hsCRP, high sensitivity C‐reactive protein; LDL‐C, low‐density lipoprotein cholesterol; T2DM, type 2 diabetes mellitus; TC, total cholesterol; TG, triglyceride.

*, ***, **** Statistically significant between the T2DM and control groups.

### 3.3 Serum IL‐36 cytokines and IL‐17 levels

**Figure 1:**
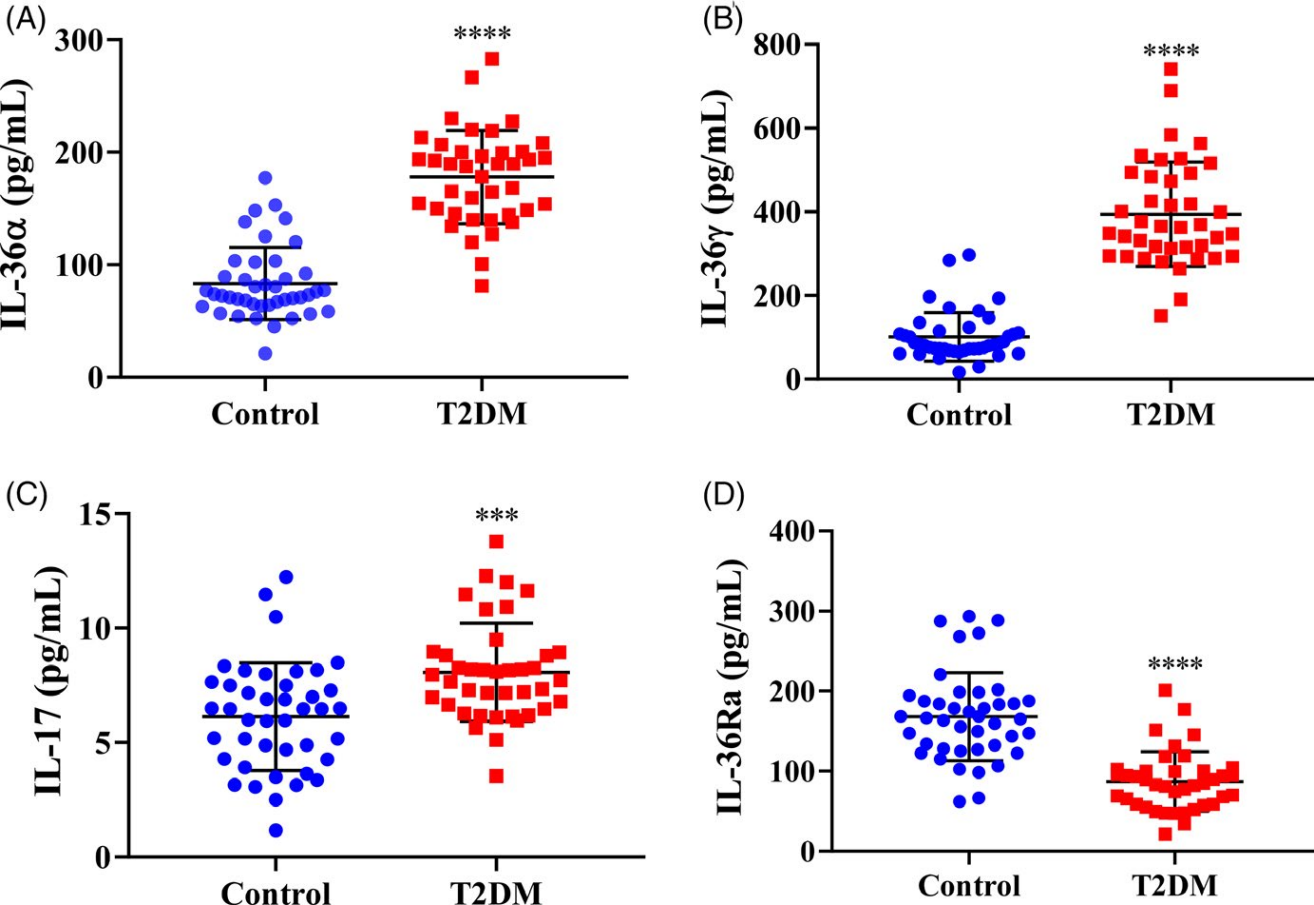
Serum IL‐36 cytokines and IL‐17 levels in T2DM patients and control subjects. A, Control (n = 42): 83.49 ± 4.26 pg/mL; T2DM (n = 40): 178 ± 5.44 pg/mL. B, Control (n = 42): 96.58 ± 6.43 pg/mL; T2DM (n = 40): 394.1 ± 16.70 pg/mL. C, Control (n = 42): 6.133 ± 0.27 pg/mL; T2DM (n = 40): 8.071 ± 0.32 pg/mL. D, Control (n = 42): 168.2 ± 7.69 pg/mL; T2DM (n = 40): 87.04 ± 5.38 pg/mL. Data are mean ± SD. ***P < .001, ****P < .0001 vs controls

We also measured the serum IL‐36 cytokines and IL‐17 levels. The IL‐36α, IL‐36γ, and IL‐17 levels in the T2DM patients were significantly higher than those in the healthy individuals (*P* < .0001, Figure 1A,B; *P* < .001, Figure 1C). Conversely, the serum IL‐36Ra level of T2DM patients was significantly lower than of the control subjects (*P* < .0001, Figure 1D).

### 3.4 Correlation of serum IL‐36 cytokines with inflammatory markers and clinical data

**Figure 2:**
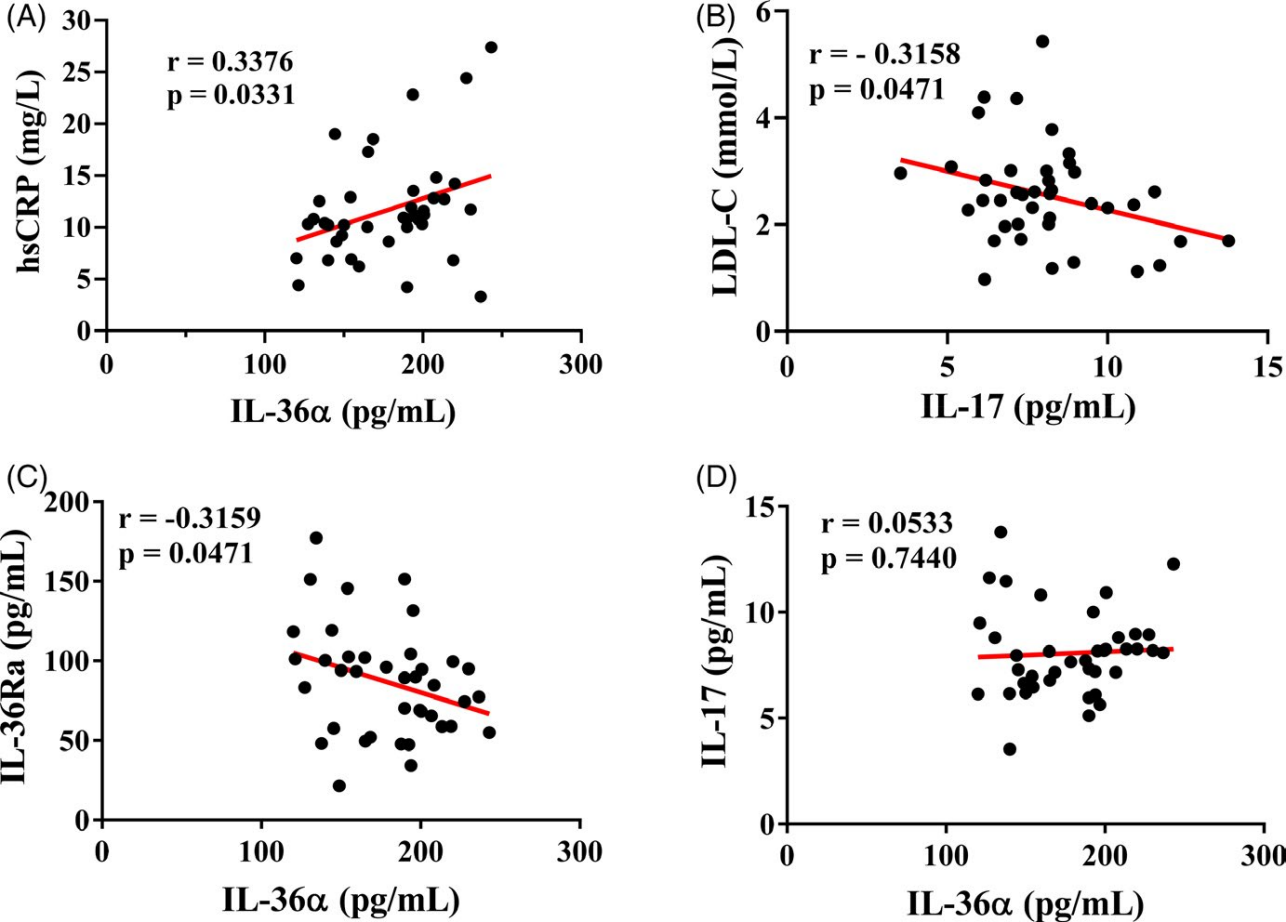
Correlation of serum IL‐36 cytokines with inflammatory markers and clinical data in T2DM patients. A, IL‐36α levels had positive correlation with hsCRP (*r* = .3376, *P* < .05). B, IL‐17 levels had negative correlation with LDL‐C (*r* = −.3158, *P* < .05). C, IL‐36α levels had negative correlation with IL‐36Ra (*r* = −.3159, *P* < .05). D, IL‐36α levels had no correlation with IL‐17 (*r* = .0533, *P* = .7440). hsCRP, high sensitivity C‐reactive protein; LDL‐C, low‐density lipoprotein cholesterol

Pearson correlation analysis of serum IL‐36 cytokines levels and clinical biochemical parameters in T2DM patients showed that IL‐36α was positively correlated with hsCRP (*r* = .3376, *P* < .05, Figure 2A). IL‐17 was negatively correlated with LDL‐C (*r* = −.3158, *P* < .05, Figure 2B). IL‐36α was also negatively correlated with IL‐36Ra (*r* = −.3159, *P* < .05, Figure 2C). However, IL‐36α was not correlated with IL‐17 (*r* = .0533, *P* > .05, Figure 2D).

## 4 DISCUSSION

This study confirmed that T2DM in patients is associated with obesity, inflammation, and IR. T2DM patients displayed increased expression of the inflammatory cytokines IL‐36α and IL‐36γ and decreased expression of the anti‐inflammatory cytokine IL‐36Ra. The inflammatory cytokine levels were directly proportional to the inflammation and blood lipid levels. Our results suggest that IL‐36 cytokines can be used as a new diagnostic indicator or target of treatment for T2DM.

The novel members of the IL‐1F (IL‐36α, IL‐36β, IL‐36γ, and IL‐36Ra) can interact with IL‐36Rs, including IL‐1Rrp2 and IL‐1RAcP. IL‐36α, IL‐36β, and IL‐36γ combine with IL‐1Rrp2 to recruit IL‐1RAcP. This causes intracellular c‐Jun N‐terminal kinase, extracellular regulated protein kinases 1/2, and NF‐κB activation, inducing increased secretion of pro‐inflammatory cytokines and chemokines, thereby promoting neutrophil aggregation, dendritic cell activation, helper T cell subgroup polarization, and keratinocyte proliferation.^6^

In recent years, IL‐36α, IL‐36β, and IL‐36γ up‐regulation in many diseases has attracted the interest of numerous researchers. These cytokines play an important role in inflammatory diseases that occur in the skin, joints, blood vessels, heart, and nerves.^7^ The current research is focused on IL‐36 in psoriasis,^6^ inflammatory arthritis,^8^ systemic lupus erythematosus,^9^ inflammatory bowel disease, including ulcerative colitis and Crohn's disease.^10^ However, as far as we know, the role of IL‐36 cytokines in T2DM is rarely reported. In this stduy, we determined the serum IL‐36α and IL‐36γ levels in T2DM patients and showed that they were significantly higher than those in normal subjects, which indicates that IL‐36α and IL‐36γ play a role in diabetes progression. Increased plasma hsCRP level is a more sensitive indicator of inflammation, which normally suggests disease progression.^11^ We found that hsCRP of T2DM patients was significantly higher than that of normal subjects and that IL‐36α was positively correlated with hsCRP, which indicates that IL‐36α is related to the inflammation progression of T2DM. The BMI index of T2DM patients in this study group was higher than that of normal subjects, which indicates that T2DM patients had a tendency to be obese. Increased fat tissue is accompanied by inflammatory reactions in obese patients, including the infiltration of macrophages and other immune cells and increased secretion of adipokines, IL‐6, MCP‐1, TNF‐α, and leptin.^12^ Inflammatory cytokines interact with the endocrine and immune systems, causing structural changes and dysfunction of pancreatic islet β cells and IR, which ultimately leads to the occurrence of T2DM.^13^ However, our data did not show a correlation between IL‐36α or IL‐36γ and FINS or HOMA‐IR.

IL‐36Ra is an IL‐36R antagonist that can inhibit IL‐36α, IL‐36β, and IL‐36γ binding to IL‐36R and, thus, has an anti‐inflammatory effect.^14^ IL‐36Ra can inhibit IL‐22 and IL‐17 production by binding to cell‐surface IL‐36R. Our results showed reduced IL‐36Ra in T2DM patients. Therefore, IL‐36Ra as an antagonist for IL‐36α, β, and γ may have an antagonistic effect on the inflammatory response of T2DM patients. Our results showed that IL‐36α is negatively correlated with IL‐36Ra, suggesting that they may play an opposite role in T2DM. Additionally, the IL‐17 level was higher in T2DM patients than in normal subjects. However, the IL‐17 level was negatively correlated with LDL, which indicates that IL‐17 was inversely associated with hyperlipidemia and obesity. We found that there was no correlation between IL‐36α and IL‐17 levels. Maybe our sample size was small and thus further research is needed. Additionally, this study did not show any correlation between IL‐36Ra and HOMA‐IR.

In conclusion, our study found that the serum levels of the inflammatory cytokines IL‐36α and IL‐36γ of T2DM patients were increased, whereas that of IL‐36Ra was decreased. Inflammatory cytokine IL‐17 levels were also increased. The inflammatory cytokine levels were directly proportional to the inflammation and blood lipid levels. The present study explored the mechanism of T2DM caused by the interaction between inflammatory cytokines, and it is expected to provide both a theoretical and experimental basis for the diagnosis and treatment of T2DM and the exploration of new drug targets.
